# Technical tips and recent development of endoscopic ultrasound‐guided choledochoduodenostomy

**DOI:** 10.1002/deo2.8

**Published:** 2021-04-21

**Authors:** Takeshi Ogura, Takao Itoi

**Affiliations:** ^1^ 2nd Department of Internal Medicine Osaka Medical College Osaka Japan; ^2^ Depaertment of Gastroenterology and Hepatology Tokyo Medical University Tokyo Japan

**Keywords:** adverse events, EUS, EUS‐CDS, EUS‐guided biliary drainage, lumen‐apposing metal stent

## Abstract

Various efforts to improve technical success rates and decrease adverse event rates have also been described in endoscopic ultrasound (EUS)‐guided choledochoduodenostomy (CDS). In particular, lumen‐apposing metal stents (LAMS) may open novel opportunities in EUS‐biliary drainage (BD). To date, various studies have been reported with EUS‐CDS using LAMS, so we should clarify the benefits and limitations of recent EUS‐CDS based on developments in both techniques and devices. In this review, we provide technical tips and describe recent developments in EUS‐CDS, along with a review of the recent literature (between 2015 and 2020). The overall technical success rate is 95.0% (939/988), and the overall clinical success rate is 97.0% (820/845). The most frequent adverse event is cholangitis or cholecystitis (24.5%, 27/110). According to previous review, pneumoperitoneum (28%, 9/34) or peritonitis associated with bile leak (23.5%, 8/34) was most commonly observed. This difference might be based on improvements in dilation devices or the use of covered metal stents. Several randomized controlled trials comparing EUS‐CDS and endoscopic retrograde cholangiopancreatography (ERCP) for malignant biliary obstruction have recently been reported. To summarize, overall technical success rates for ERCP and EUS‐CDS were 92.7% (101/109) and 91.1% (72/79), respectively (*p* = 0.788). Overall clinical success rates for ERCP and EUS‐CDS were 94.1% (96/102) and 93.6% (72/78), respectively (*p* = 0.765). Further high‐quality evidence is needed to establish EUS‐CDS as a primary drainage technique.

## INTRODUCTION

The gold‐standard treatment technique for bile duct obstruction is stent deployment under endoscopic retrograde cholangiopancreatography (ERCP) guidance, and technical success is usually obtained in most cases. However, failed biliary cannulation is observed in up to 10% of cases.^1^ Therefore, in cases of failed biliary cannulation, alternative access methods are needed. Percutaneous transhepatic biliary drainage (PTBD) is traditionally attempted as an alternative drainage method, but adverse event rates are reportedly relatively high.^2^, ^3^ In addition, PTBD is an external drainage method, so quality of life may be decreased. Recently, clinical impacts of endoscopic ultrasound (EUS) have been reported with diagnostic modalities such as fine‐needle aspiration (FNA) and treatment modalities such as biliary drainage (BD). EUS‐BD can be divided into two main approach routes: trans‐gastric or trans‐duodenal. As representative procedures, EUS‐guided hepaticogastrostomy (HGS) can be performed via the trans‐gastric route, while EUS‐guided choledochoduodenostomy (CDS) can also be performed via the trans‐duodenal route. EUS‐BD is indicated for patients who failed ERCP. Normally, if duodenum approach can be performed, EUS‐CDS may be selected as alternative method. On the other hand, if patients who are complicated with duodenal obstruction or surgically altered anatomy, EUS‐HGS is selected. In addition, EUS‐guided antegrade stenting (AG) or EUS‐HGS combined with AG is also developed. However, most suitable indication for these procedures is still unclear; therefore, further comparison studies are needed.

Although adverse event rates have frequently been reported in EUS‐BD, various efforts to improve technical success rates and decrease adverse event rates have also been described. In particular, lumen‐apposing metal stents (LAMS) may open novel opportunities in EUS‐BD. Because the intrahepatic bile duct is not overly dilated and the saddle length is short, LAMS may not be suitable in EUS‐HGS procedures. On the other hand, various studies have been reported with EUS‐CDS using LAMS, so we should clarify the benefits and limitations of recent EUS‐CDS based on developments in both techniques and devices.

In this review, we provide technical tips and describe recent developments in EUS‐CDS, along with a review of the recent literature.

## OVERVIEW OF RECENT CLINICAL OUTCOMES OF EUS‐CDS

Table 1 shows recent studies regarding EUS‐CDS (published in the last 5 years [between 2015 and 2020], and including over 15 cases).^4^, ^5^, ^6^, ^7^, ^8^, ^9^, ^10^, ^11^, ^12^, ^13^, ^14^, ^15^, ^16^, ^17^, ^18^, ^19^, ^20^, ^21^, ^22^, ^23^, ^24^, ^25^ The main indication is failed ERCP, but recent studies have tried to evaluate the clinical efficacy of EUS‐CDS for primary drainage.^8^, ^13^, ^19^, ^20^, ^26^ Technical success rates of EUS‐CDS range from 88.8% to 100%, and the clinical success rate ranges from 85.5% to 100%. Further, the overall technical success rate is 95.0% (939/988), and the overall clinical success rate is 97.0% (820/845). Based on comparisons of technical and clinical success rates between recent and previous data (technical success, 93% [199/213]; clinical success, 98% [183/187]) in reviews from 2003 to 2014,^27^ satisfactory results may have already been obtained.

**TABLE 1:** Recent studies of EUS‐CDS reported in the last 5 years and including over 15 cases

Author/year	Number of patients	Stent types (product name)	Procedure time (min)	Technical success rate, % (*n*)	Clinical success rate, % (*n*)	Short‐term adverse events, %(including postprocedural adverse events)	Long‐term adverse event rate, %(including stent dysfunction)
Teoh/2020^4^	26	LAMS (Niti‐S Spaxus)	40.4	88.5 (23/26)	88.9 (24/27)	11.5 (mis‐deployment, *n* = 3)	7.7 (blocked stent, *n* = 2)
de Benito Sanz/2020^5^	37	LAMS (AXIOS, Hot AXIOS)	ND	100 (37/37)	94.7 (36/37)	10.8 (cholangitis, *n* = 1; cholecystitis, *n* = 1; bleeding, *n* = 2; other, *n* = 1)	ND
de Benito Sanz/2020^5^	20	SEMS (Hanarostent, Bona stent, Wallflex)	ND	100 (20/20)	100 (20/20)	20.0 (cholangitis, *n* = 1; cholecystitis, *n* = 1; bile leakage, *n* = 1; other, *n* = 1)	ND
Chin/2020^6^	56	LAMS (Hot AXIOS)	ND	100 (56/56)	ND	ND	ND
Ogura/2020^7^	22	SEMS (Bona stent, EGIS double bare)	12.6 (median)	100 (22/22)	100 (22/22)	9.0 (abdominal pain, *n* = 2)	18.2 (stent kinking, *n* = 3; stent dislocation, *n* = 1)
Kuroka/2020^8^	92	SEMS (Bona stent, Wallflex, X‐suit NIR)	17.5 (median)	92.8 (83/92)	91.6 (76/83)	10.0 (cholangitis, *n* = 5; peritonitis, *n* = 2; bleeding, *n* = 1; double penetration of duodenum, *n* = 1)	3.0 (cholecystitis, *n* = 2; liver abscess, *n* = 1)
Matsumoto/2020^9^	151	Plastic stent (Tannenbaum, Flexima) SEMS (Wallflex, X‐Suit NIR)	ND	96.5 (137/142)	98.5 (135/137)	20.4 (peritonitis, *n* = 14; early stent dysfunction, *n* = 9; cholangitis, *n* = 2; bleeding, *n* = 2; cholecystitis, *n* = 1; stent migration, *n* = 1)	2.1 (cholecystitis, *n* = 3)
Jacques/2020^10^	70	LAMS (Hot AXIOS)	5 (mean)	98.6 (69/70)	98.6 (69/70)	3.0 (bleeding, *n* = 1; stent in situ, *n* = 1)	10.0 (tumor obstruction, *n* = 4; jaundice due to migration, *n* = 1; cholangitis due to bezoar, *n* = 1; bacteremia, *n* = 1)
El Chafic/2019^11^	67	LAMS (Hot AXIOS)	27.6 (mean)	95.5 (64/67)	100 (40/40)	7.8 (bleeding, *n* = 1; abdominal pain, *n* = 2; peritonitis, *n* = 1)	17.5 (recurrent biliary obstruction, *n* = 7)
Minaga/2019^12^	23	SEMS (modified Niti‐S)	30.5 (mean)	82.6 (19/23)	95.7 (22/23)	8.7 (cholecystitis, *n* = 2)	8.7 (stent occlusion, *n* = 1; stent migration, *n* = 1)
Nakai/2019^13^	34	SEMS (WallFlex)	25 (median)	97.1 (33/34)	100 (34/34)	12.0 (abdominal pain, *n* = 2; cholangitis, *n* = 1; cholecystitis, *n* = 1)	29 (migration, *n* = 6; sludge/food impaction, *n* = 3; stent impaction to DU, *n* = 1)
Itonaga/2019^14^	20	SEMS (BileRush)	19.8 (mean)	95.0 (19/20)	100 (19/19)	5 (peritonitis, *n* = 1)	21.1 (stent dysfunction, *n* = 4)
Jacques/2019^15^	52	LAMS (Hot AXIOS)	ND	88.5 (46/52)	100 (46/46)	3.8 (cholangitis, *n* = 1; bleeding, *n* = 1)	13.5 (tumor obstruction, *n* = 4; sump syndrome, *n* = 2; stent migration, *n* = 1)
Anderloni/2019^16^	46	LAMS (Hot AXIOS)	14.7 (mean)	93.5 (43/46)	97.1 (42/43)	11.6 (fatal bleeding, *n* = 1; stent occlusion due to food impaction, *n* = 3; spontaneous stent migration, *n* = 1)	2.3 (stent obstruction due to food impaction, *n* = 1)
Rai/2018^17^	30	SEMS (Wallflex)	30 (median)	93.3 (28/30)	100 (28/28)	10.0 (bile leak, *n* = 1; hemobilia, *n* = 1; stent block, *n* = 1)	ND
Tsuchiya/2018^18^	19	LAMS (Hot AXIOS)	16.2 (mean)	100 (19/19)	95.0 (18/19)	10.5 (acute cholangitis, *n* = 2)	21.1 (stent occlusion due to food impaction, *n* = 2; tumor obstruction, *n* = 1; stent dislocation, *n* = 1)
Paik/2018^19^	33	SEMS (DEUS)	5.8 (median)	90.6 (29/32)	87.5 (28/32)	6.3 (ND)	9.4 (ND)
Bang/2018^20^	33	SEMS (Viabil)	24.2 (mean)	90.9 (30/33)	97.0 (32/33)	14.7 (abdominal pain, *n* = 5; cholecystitis, *n* = 1; bile peritonitis, *n* = 1)	21.2 (ND)
Lu/2017^21^	17	SEMS (Wallflex)	35.9 (mean)	100 (17/17)	100 (17/17)	11.8 (hemorrhage, *n* = 2)	ND
Cho/2017^22^	33	SEMS (Hybrid)	20 (median)	100 (33/33)	100 (33/33)	15.1 (pneumoperitoneum, *n* = 1; bleeding, *n* = 1; cholangitis, *n* = 3)	15.1 (stent occlusion, *n* = 5)
Kunda/2016^23^	57	LAMS (Hot AXIOS)	22.4 (mean)	98.2 (56/57)	96.4 (54/56)	7 (duodenal perforation, *n* = 2; bleeding, *n* = 1; cholangitis, *n* = 1)	9.3 (stent migration, *n* = 1; sump syndrome, *n* = 4)
Khashab/2016^24^	60	Metal, Plastic	51.0 (mean)	93.3 (56/60)	85.5 (ND)	13.3 (peritonitis, *n* = 1; bile leak, *n* = 1; cholangitis, *n* = 1; bleeding, *n* = 1; pancreatitis, *n* = 2; perforation, *n* = 1; pneumoperitoneum, *n* = 1)	13.3 (stent occlusion, *n* = 5; stent migration, *n* = 3)
Kwakubo/2016^25^	26	SEMS (WallFlex)	19.7 (mean)	ND	96.2 (25/26)	26.9 (cholecystitis, *n* = 3; liver abscess, *n* = 2; peritonitis, *n* = 1; cholangitis, *n* = 1)	

Abbreviations: DU, duodenum; EUS‐CDS, endoscopic ultrasound‐guided choledochoduodenostomy; LAMS, lumen‐apposing metal stent; ND, not described; SEMS, self‐expandable metal stent.

On the other hand, the overall early adverse event rate was 12.2% (110/900), lower than described from previous data (16%, 34/213), although significant difference was not observed (*p* = 0.09). EUS‐CDS has the potential to cause several adverse events, including (1) infection (peritonitis, cholangitis, cholecystitis); (2) pneumoperitoneum; (3) bile leakage, biloma; (4) bleeding; (5) abdominal pain; (6) perforation; (7) stent migration; and (8) double mucosal puncture. The most frequent adverse event is cholangitis or cholecystitis (24.5%; 27/110) in recent data. On the other hand, according to previous data, pneumoperitoneum (28%, 9/34) or peritonitis associated with bile leak (23.5%, 8/34) was most commonly observed. This difference might be based on improvements in dilation devices or the use of covered metal stents. Indeed, Matsumoto et al. analyzed peritonitis as an adverse event during EUS‐CDS, and concluded that plastic stents were a risk factor for peritonitis.^9^ That study enrolled 151 patients who underwent EUS‐CDS. Early adverse events were observed in 29 patients, with peritonitis observed in 14 patients (9.9%). In univariate analysis, plastic stent deployment was a significant factor compared with deployment of a covered self‐expandable metal stent (SEMS) (odds ratio [OR] 3.69; 95% confidence interval [CI] 1.15–11.8; *p* = 0.043). Multivariate analysis showed an OR of 4.13 (95% CI 1.13–16.39; *p* = 0.03). Covered SEMS may thus be suitable in EUS‐CDS compared with plastic stents.

## TECHNICAL REVIEWS OF EUS‐CDS

### Bile duct puncture and guidewire deployment

The echoendoscope is inserted into the duodenum, and the common bile duct is identified in a long position. The common bile duct is then punctured using a 19‐G needle under color Doppler guidance to avoid injury to blood vessels. Before bile duct puncture, we should pay attention to two important issues.

**FIGURE 1:**
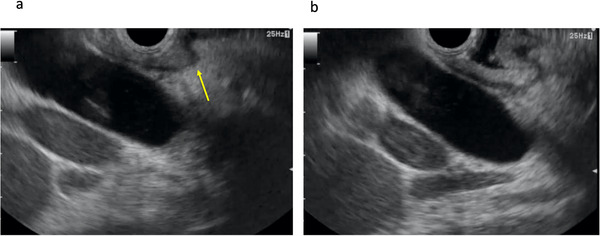
Double mucosal sign. (a) Double mucosal sign is observed (arrow). (b) After water injection, echo‐free space is obtained

First, to prevent double mucosal puncture, careful monitoring for the double mucosal sign should be performed (Figure 1a). If the common bile duct is punctured in this situation, double mucosal puncture may occur. Bleeding or perforation might occur if double mucosal puncture is complicated.^27^ To prevent this adverse event, the water‐filling technique may be useful (Figure 1b).^28^ Matsumoto et al. described risk factors for double mucosal puncture during EUS‐CDS.^9^ In that study, double mucosal puncture occurred significantly more frequently with oblique‐viewing echoendoscopes (7.0%) compared with forward‐viewing echoendoscopes (0.0%; *p* = 0.024). Because a forward‐viewing echoendoscope can adhere closely to the puncture site without catching on the duodenum membrane, Matsumoto et al. recommended attempting EUS‐CDS using a forward‐viewing echoendoscope. However, indications for procedures using forward‐viewing echoendoscopes are less frequent than for those using oblique‐viewing echoendoscopes, and this scope is thus unavailable in many institutes. Further evaluations of favorable techniques to prevent this adverse event are needed.

**FIGURE 2:**
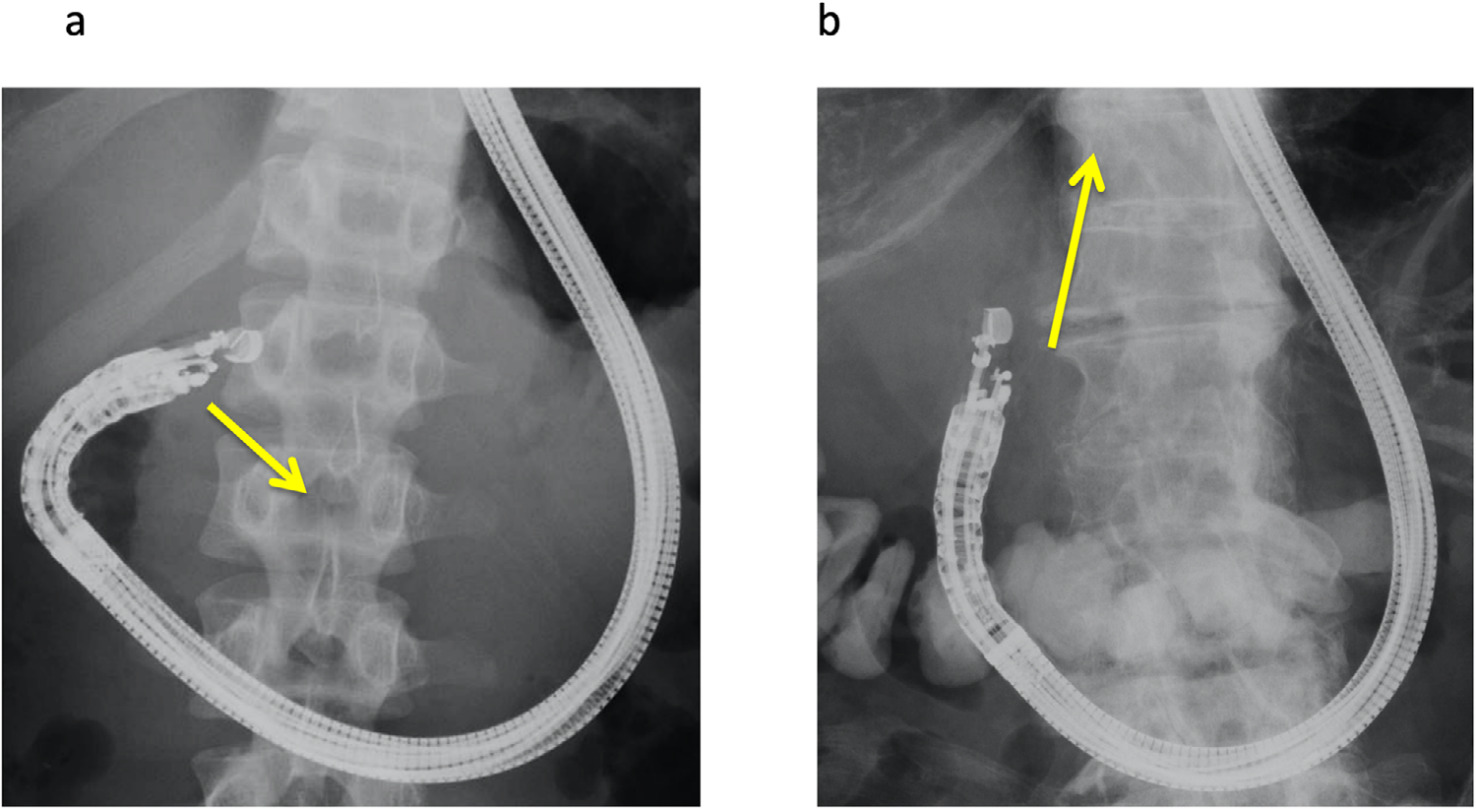
Common bile duct puncturing. If the common bile duct is punctured as shown (a), the guidewire may be easily advanced toward the ampulla of Vater. On the other hand, if the common bile duct is punctured as shown (b), the guidewire may be easily advanced into the intrahepatic bile duct

Second, we should pay attention to not only EUS imaging, but also fluoroscopic imaging. After common bile duct puncture, a guidewire should be advanced into the intrahepatic bile duct. On EUS imaging, when the CBD is aligned parallel to the FNA needle, the guidewire can be easily advanced toward the hepatic hilum.^27^ However, if the common bile duct is punctured as shown in Figure 2a, the guidewire may be easily advanced toward the ampulla of Vater. On the other hand, if the common bile duct is punctured as shown in Figure 2b, the guidewire may be easily advanced into the intrahepatic bile duct. Echoendoscope shape should thus be checked before bile duct puncture under fluoroscopic imaging. As a novel device, Ryou et al. reported the initial experience with a steerable access device during EUS‐BD.^29^ Interestingly, after stylet removal, the blunt‐tipped access catheter assumes a predetermined curvature (90° or 135°) and is fully rotatable. In addition, wire shearing can be avoided because of the blunt tip and the coaxial alignment with the wire relative to the catheter tip. In this study, 22 patients underwent EUS‐BD using this novel device, including seven EUS‐CDS cases. As a result, after bile duct puncture, rotation of the access system and selective wire advancement in the preferred direction were successful in all patients. This device may be clinically useful, although further evaluation is needed. After bile duct puncture, guidewire deployment is attempted. In almost all reports, a 0.025‐inch guidewire was deployed. Although evidence remains lacking, deployment of a stiff‐type guidewire or double guidewire may be preferred over insertion of various devices and stabilization of the echoendoscope by the guidewire. Shiomi et al. described the clinical benefits of double guidewire deployment.^30^ In that report, they mentioned that the double guidewire facilitates stability of the echoendoscope position, improved visualization of the guidewire under EUS imaging, easy insertion of devices, and safe guidewire roles. Guidewires may thus play important roles in stabilizing the echoendoscope itself, not only in EUS‐HGS but also in EUS‐CDS. However, further comparative studies are needed between single and double or between soft and stiff guidewires. Finally, no evidence has been accumulated regarding guidewire deployment site (left or right intrahepatic bile duct).

### Tract dilation

**FIGURE 3:**
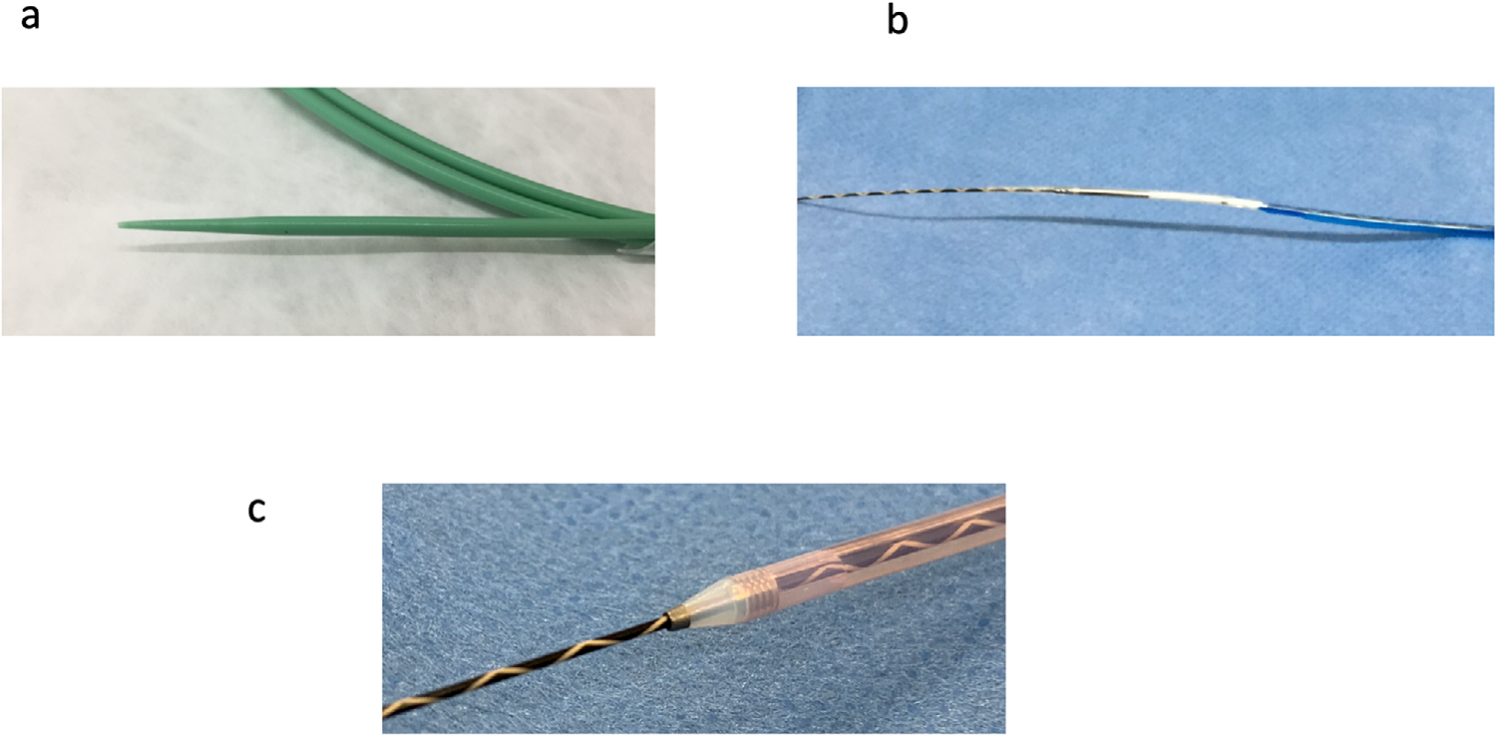
Novel dilation devices. (a) ES dilator (Zeon Medical Co., Tokyo, Japan). (b) REN biliary dilation catheter (KANEKA, Osaka, Japan). (c) Fine 025 (Medico's Hirata Inc., Osaka, Japan)

In stent deployment, dilation of the common bile duct and duodenum wall is needed. During EUS‐BD, bile leakage can occur after fistula dilation. Procedural steps during fistula dilation should thus be minimized. Fistula dilation should be certainly performed in a single step. Novel dilation devices are now available, such as an ultra‐tapered mechanical dilator,^31^ a fine‐gauge balloon catheter,^32^ and a fine‐gauge electrocautery dilator^33^ (Figure 3a–c). Honjo et al. compared use of an ultra‐tapered mechanical dilator in 26 patients with use of an electrocautery dilator in 23 patients during EUS‐HGS.^31^ Technical success rates did not differ significantly between groups (100% [23/23] vs. 92.3% [24/26]; *p* = 0.52). Overall adverse event rates also differed significantly between groups (30.4% [7/23] vs. 15.3% [4/26]; *p* = 0.35). However, bleeding was significantly more frequent in the electrocautery dilator group (*p* = 0.04). Although EUS‐CDS cases were not included, technical tips are similar. This device may thus be useful for EUS‐CDS procedures. Also, in that study, the authors noted that electrocautery dilation can cause burning effects to the hepatic parenchyma and vessels around the needle tract or gastrointestinal lumen, causing unexpected bleeding or inflammation. To avoid such effects, a novel fine‐gauge electrocautery dilator has been developed.^33^ Compared with conventional electrocautery dilators, the burning effects are smaller, so this device may be clinically safe. On the other hand, Amano et al. conducted a prospective study of EUS‐BD using a fine‐gauge balloon catheter.^32^ A total of 20 patients were enrolled in that study, and technical success was obtained in each. Among this group, 11 patients underwent EUS‐CDS with a short procedure time (median 11 min; range 8–16 min) without any severe adverse events. In terms of dilation devices, evidence remains lacking regarding whether dilation devices should be used, such as electrocautery or nonelectrocautery. The most favorable dilation device should be determined in a prospective randomized controlled trial. Even if these novel devices are used, bile leakage can occur after fistula dilation. In addition, compared with EUS‐HGS, no tamponade effects such as with hepatic parenchyma are seen during EUS‐CDS.^34^ Bile leakage from the fistula is thus more likely in EUS‐CDS compared with EUS‐HGS. Ultimately, insertion of a stent delivery system without fistula dilation may be ideal, such as electrocautery‐enhanced LAMS. Indeed, according to previous reports of EUS‐CDS using electrocautery‐enhanced LAMS,^4^, ^5^, ^10^, ^11^, ^15^, ^16^, ^18^, ^23^ bile peritonitis and leakage have not been reported as procedure‐related adverse events. Electrocautery‐enhanced LAMS thus offers theoretical advantages in preventing this adverse event, because this system allows a single‐step procedure, although further comparative studies between conventional SEMS and LAMS are needed to confirm such advantages.

### Stent deployment

After the common bile duct and duodenum wall are dilated, insertion of the stent delivery system is performed. Stent release is then carefully started from the common bile duct. Under such circumstances, performing this procedure with visual confirmation under EUS imaging may be important to prevent misplacement. Also, to prevent stent migration into the abdominal cavity, an intrascope channel‐release technique may be useful.^35^

**FIGURE 4:**
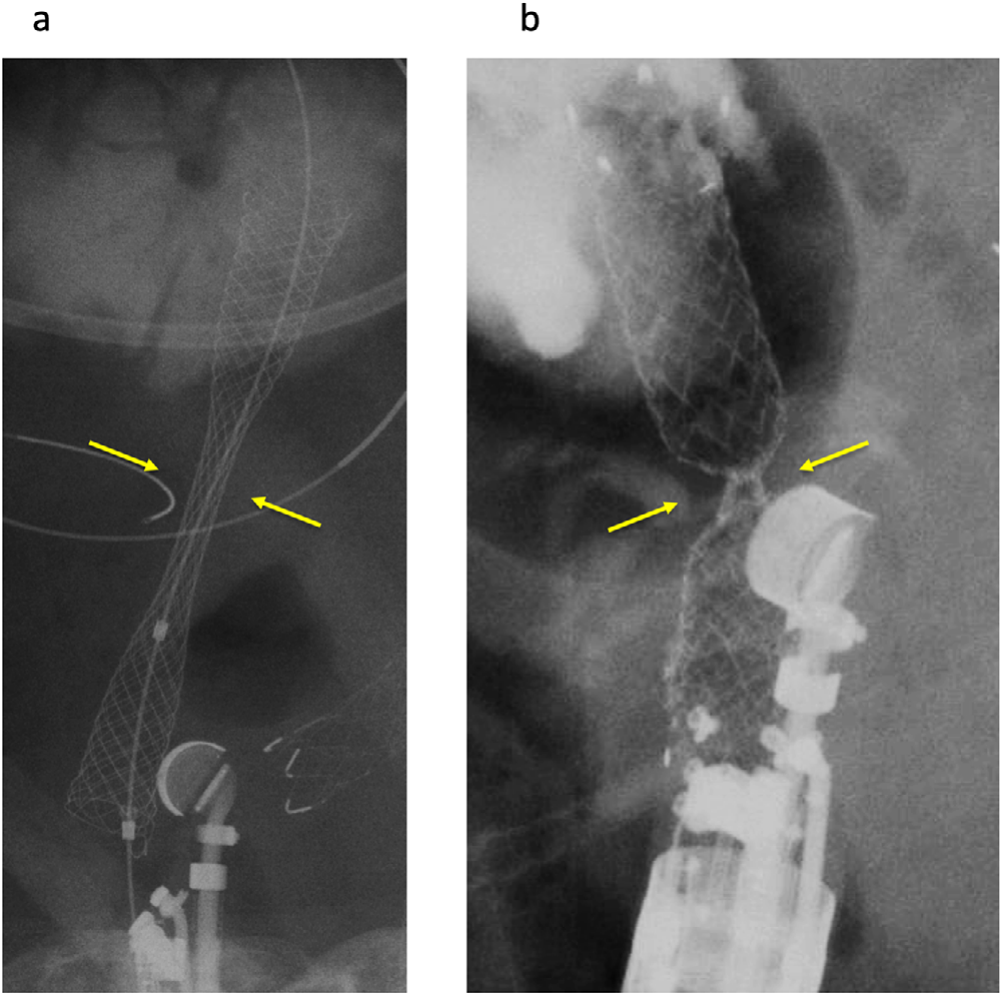
Comparison between braided type and laser‐cut type in transluminal stenting. Because radial force is smaller than with the braided type (a, arrow), a deep notch is formed after stent deployment (b, arrow)

Stent selection is an important factor to prevent adverse events. Plastic stents, SEMS or LAMS can be selected as EUS‐CDS stents. As noted above, plastic stents carry a risk of bile leakage or peritonitis. If a large fistula is created before stent placement, bile leakage from the gap between the stent and fistula is likely to occur because of the fine gauge of the plastic stent. Therefore, according to the recent studies shown in Table 1, most endoscopists select SEMS or LAMS as EUS‐CDS stents. Fully covered SEMS (FCSEMS) are available in two forms: braided or laser cut. Figures 4a and 4b show comparative images of braided and laser‐cut stents after transluminal stenting. From the perspective of preventing stent migration during stent deployment, the laser‐cut type may be suitable. Because radial force is smaller than with the braided type, a deep notch is formed after stent deployment.^9^ However, this theory should be confirmed in a comparative study, because evidence for which stent types are optimal for use as EUS‐CDS stents remains lacking.

Several novel covered SEMS, which are focused on preventing bile peritonitis, stent kinking, and stent migration, have recently been reported. Itonaga et al. evaluated EUS‐CDS using a thin stent delivery system in a multicenter prospective study.^14^ In that study, a novel, laser‐cut SEMS with a 7.5‐Fr delivery system was used as the EUS‐CDS stent, and the stent delivery tip was tapered. They reported that the thinness of the delivery system may facilitate successful insertion without fistula dilation as an advantage of this stent. However, in fact, the stent delivery system without fistula dilation was successful in only 31.6% (6/19). They also discussed the need for stent improvements such as a thinner and more tapered tip. Stent migration is one critical limitation of EUS‐CDS if FCSEMS is selected. Cho et al. conducted a study of EUS‐CDS using a new hybrid stent.^22^ The distal portion of this stent, which is 3.5‐cm long, is composed of silicone‐covered nitinol wire to prevent bile leakage. The covered portion shows proximal and distal anchoring flaps to immobilize the stent and prevent proximal and distal stent migration. In this study, EUS‐CDS was attempted in 33 patients. As a result, technical and clinical success was obtained in all patients with a short procedure time (median, 20 min). In addition, stent migration was not observed in any patients. Moreover, after long‐term follow‐up (median, 148.5 days), no stent migration was observed. This anchoring flap system may thus act to prevent stent migration. From the perspective of one factor associated with early stent dysfunction, axial force, which can cause stent kinking between the stent and common bile duct, may result.^27^ A novel SEMS has been developed with low axial force (double‐bared stent). We previously conducted comparisons between FCSEMS and double‐bared stents during EUS‐CDS.^7^ Among 22 patients, stent dysfunction occurred in five patients (FCSEMS, *n* = 4; double‐bared stent, *n* = 1) and was associated mainly with cholangitis. Kinking caused cholangitis in three FCSEMS patients treated by stent exchange. Stent selection may thus be an important factor to prevent this adverse event.

As a recent expansion to the indications for LAMS, various reports of EUS‐CDS using LAMS have been reported. LAMS was first reported by Binmoeller and Shah in 2011.^36^ This conventional LAMS has facilitated the technical improvement of EUS‐guided transluminal stenting. LAMS facilitates adhesion between the target lesion and gastrointestinal lumen with high appositional force. Stent migration during stent deployment can thus be prevented. More recently, LAMS with an electrocautery tip has been developed. This device has allowed for innovative changes in techniques, such as insertion of the stent delivery system without fistula dilation.^16^ This fact may play important roles such as decreasing adverse events associated with bile leakage, and reducing procedure time and fluoroscopy exposure time.^37^, ^38^, ^39^ This stent uses a relatively large, stiff delivery system and a new stent‐release system, so stent deployment can be technically demanding.^23^ Indeed, the utility of LAMS with a conventional SEMS delivery system has been reported.^4^, ^40^ Jacques et al. evaluated EUS‐CDS using electrocautery‐enhanced LAMS based on operator experiences with EUS‐BD.^10^ In this study, EUS‐CDS was performed by 29 experts and 23 nonexperts. No significant difference between the two groups was seen in terms of technical success rates (82.8% vs. 95.7%; *p* = 0.21), clinical success rates (96.6% vs. 100%; *p* > 0.99), and mean procedure time (9.9 min vs. 10.6 min; *p* = 0.85). This result may encourage endoscopists, even if they are unfamiliar with the use of the LAMS system. Despite investigating the first US experience, El Chafic et al. reported clinical results of EUS‐CDS using LAMS with a high technical success rate (95.5%, 64/67) and low adverse event rate (6.3%, 4/64).^11^ In this study, technical success was not obtained in two patients because of mispositioning of LAMS. Also, inability to puncture electrocautery enhanced LAMS delivery system into the bile duct, which was suspected tangential entry tract to the bile duct, was observed in one patient.

As described above, LAMS appear to be favorable stents in EUS‐CDS procedures. However, because the proximal site is open toward the gastric side, food impaction or residue may easily occur. Matsumoto et al. compared SEMS direction to the oral or anal side in EUS‐CDS.^9^ In this study, stent direction to the oral side represented an independent risk factor for early stent dysfunction (OR 43.47; 95% CI 6.21–304.32; *p *< 0.001) in multivariate analysis. They also reported that when the distal side of the stent faces the oral side, food residue can enter the stent and lead to early stent dysfunction as a complication. On the other hand, de Benito Sanz et al. recently conducted a comparison between LAMS and SEMS. Although the direction of SEMS was not described in that study, no significant difference between groups was seen regarding adverse events, reinterventions, or survival rates. Whether the direction of EUS‐CDS stent has real‐world influences in clinical practice should be evaluated in future prospective comparative studies.

## EUS‐CDS VERSUS ERCP AS FIRST‐LINE DRAINAGE METHODS

EUS‐CDS is currently indicated for patients with failed ERCP or inaccessible papilla due to duodenal obstruction.^27^ Compared with ERCP, a critical advantage of EUS‐CDS is the theoretical absence of any risk of postprocedural acute pancreatitis. In addition, recent developments in various devices have facilitated the next steps in EUS‐CDS, such as primary drainage rather than merely an alternative option. For the first clinical trial of EUS‐CDS as primary drainage, Hara et al. conducted a prospective feasibility study of EUS‐CDS using plastic stents for malignant lower bile duct obstruction.^41^ In that study, technical and clinical success rates were 94% (17/18) and 100% (17/17), respectively. Procedure‐related adverse events were encountered in three patients (17%). Nakai et al. also conducted a prospective feasibility study of EUS‐CDS using SEMS in a multicenter setting.^13^ Thirty‐four patients were enrolled in the study, with SEMS successfully deployed in 33 patients (97%), while one patient underwent EUS‐CDS using a plastic stent because of failed insertion of the SEMS delivery system into the common bile duct. Median procedure time was 25 min, and clinical success was obtained in all patients. In addition, although adverse events were observed in 15% of cases (*n* = 5), acute pancreatitis was not seen in any patient.

Several randomized controlled trials comparing EUS‐CDS and ERCP for malignant biliary obstruction have recently been reported (Table 2).^19^, ^20^, ^26^ To summarize, overall technical success rates for ERCP and EUS‐CDS were 92.7% (101/109) and 91.1% (72/79), respectively (*p* = 0.788). Overall clinical success rates for ERCP and EUS‐CDS were 94.1% (96/102) and 93.6% (72/78), respectively (*p* = 0.765). In addition, acute pancreatitis was only observed in the ERCP group. According to a multicenter randomized trial by Paik et al.,^19^ which included EUS‐HGS cases, although technical and clinical success rates were comparable between groups, overall adverse events including acute pancreatitis (ERCP vs. EUS‐BD, 14.8% vs. 0%) were significantly more frequent in the ERCP group (19.7%) than in the EUS‐CDS group (6.3%; *p* = 0.03). In addition, stent patency at 6 months was higher in EUS‐BD (85.1% vs. 48.9%; *p* = 0.001) and mean stent patency was longer (208 days vs. 165 days). Interestingly, EUS‐BD was associated with greater preservation of quality of life after 12 weeks of biliary drainage. The authors therefore concluded that EUS‐BD may be superior in terms of lower adverse event rates and preserved quality of life. According to a recent meta‐analysis that included retrospective studies,^42^ the technical success rate was 94.73% in the ERCP group and 93.67% in the EUS‐CDS group (OR 1.2, 95% CI 0.44–3.24; *p* = 0.72). Clinical success rates were also similar in the ERCP and EUS‐CDS groups (94.21% vs. 91.23%; OR 1.44, 95% CI 0.63–3.29; *p* = 0.66). Although procedure‐related adverse event rates did not differ significantly between ERCP and EUS‐CDS groups (15.2% vs. 22.3%; OR 1.59, 95% CI 0.89–2.84; *p* = 0.40), pancreatitis was observed only in the ERCP group (9.5%). EUS‐CDS may thus be worth considering for primary drainage, especially from the perspective of avoiding postprocedural pancreatitis.

**TABLE 2:** Randomized controlled trials comparing EUS‐CDS and ERCP for primary drainage

Author/year	Method	Number of patients, *n*	Technical success rate, % (*n*)	Clinical success rate, % (*n*)	Procedure time, min	Adverse event rate, %	Stent patency (mean, days)
Paik^a^/2018^19^	ERCP EUS‐CDS	64 33	90.2 (55/61) 90.6 (29/32)	94.5 (52/55) 87.5 (28/32)	11 (median) 5^b^ (median)	19.7 6.3	165 208
Bang/2018^20^	ERCP EUS‐CDS	34 33	94.1 (32/34) 90.9 (30/33)	91.2 (31/34) 97.0 (32/33)	21 (median) 25 (median)	14.7 21.2	170 182
Park/2018^26^	ERCP EUS‐CDS	14 14	100 (14/14) 92.8 (13/14)	92.8 (13/14) 100 (13/13)	31 (mean) 43 (mean)	0 0	403 379

Abbreviations: BD, biliary drainage; ERCP, endoscopic retrograde cholangiopancreatography; EUS‐CDS, endoscopic ultrasound‐guided choledochoduodenostomy; ND; not described.

^a^ Among EUS‐BD, hepaticogastrostomy (HGS) patients are excluded.

^b^ Including EUS‐HGS cases.

On the other hand, Bang et al. also evaluated EUS‐CDS and ERCP as primary drainage for malignant biliary obstruction due to pancreatic cancer in a randomized controlled trial.^20^ In that study, technical success (ERCP vs. CDS: 94.1% vs. 90.9%; *p* = 0.637), clinical success (ERCP vs. CDS: 91.2% vs. 97.0%; *p* = 0.614), and median procedure time (ERCP vs. CDS: 21 min vs. 25 min; *p* = 0.178) showed no significant differences between groups. In addition, adverse events (ERCP vs. CDS: 21.2% vs. 14.7%; *p* = 0.490) were similar between groups. They therefore concluded both procedures were similar in clinical practice. Park et al. conducted a randomized controlled trial and concluded that EUS‐CDS was not superior to ERCP in terms of relief from malignant biliary obstruction.^26^ Characteristically, although EUS‐CDS may show a lower frequency of tumor ingrowth compared with transpapillary stenting, stent dysfunction due to food impaction or stent migration was frequently observed in EUS‐CDS. Therefore, although EUS‐CDS may have potential for use as a first‐line drainage method in place of ERCP, stricter evidence from a large‐scale randomized trial is needed.

## CONCLUSIONS

Electrocautery‐enhanced LAMS can be deployed without fistula dilation, which may reduce adverse event rates associated with bile leakage. In addition, several studies have shown the potential of primary drainage methods for malignant biliary obstruction. However, further high‐quality evidence is needed to establish EUS‐CDS as a primary drainage technique.

## CONFLICT OF INTEREST

The authors declare that there is no conflict of interest.

## FUNDING INFORMATION

None.
